# 20-State Molecular Switch in a Li@C_60_ Complex

**DOI:** 10.1021/acsomega.3c01455

**Published:** 2023-05-25

**Authors:** Ali K. Ismael

**Affiliations:** † Department of Physics, Lancaster University, Lancaster LA1 4YB, U.K.; ‡ Department of Physics, College of Education for Pure Science, Tikrit University, Salahuddin, Al-Qadissiya street 34001, Tikrit, Iraq

## Abstract

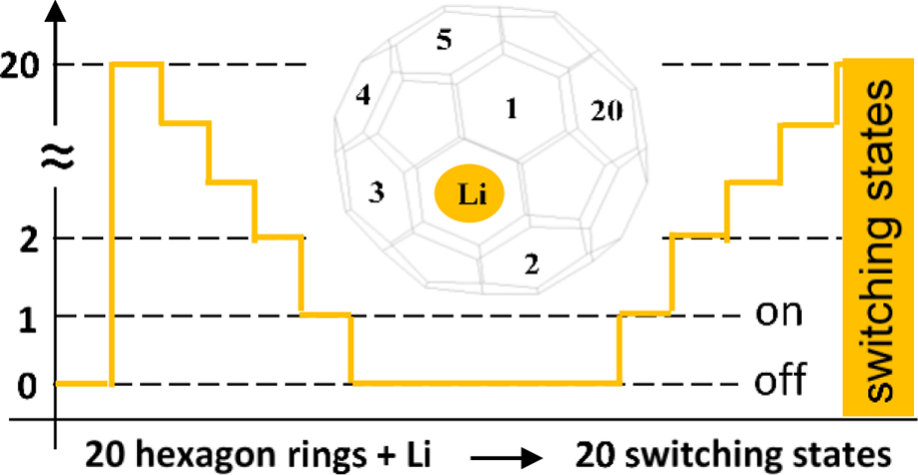

A substantial potential advantage of industrial electric and thermoelectric devices utilizing endohedral metallofullerenes (EMFs) is their ability to accommodate metallic moieties inside their empty cavities. Experimental and theoretical studies have elucidated the merit of this extraordinary feature with respect to developing electrical conductance and thermopower. Published research studies have demonstrated multiple state molecular switches initiated with 4, 6, and 14 distinguished switching states. Through comprehensive theoretical investigations involving electronic structure and electric transport, we report 20 molecular switching states that can be statistically recognized employing the endohedral fullerene Li@C_60_ complex. We propose a switching technique that counts on the location of the alkali metal that encapsulates inside a fullerene cage. The 20 switching states correspond to the 20 hexagonal rings that the Li cation energetically prefers to reside close to. We demonstrate that the multiswitching feature of such molecular complexes can be controlled by taking advantage of the off-center displacement and charge transfer from the alkali metal to the C_60_ cage. The most energetically favorable optimization suggests 1.2–1.4 Å off-center displacement, and Mulliken, Hirshfeld, and Voronoi simulations articulate that the charge migrates from the Li cation to C_60_ fullerene; however, the amount of the charge transferred depends on the nature and location of the cation within the complex. We believe that the proposed work suggests a relevant step toward the practical application of molecular switches in organic materials.

## 1 Introduction

Molecular electronics field existed to fulfill the desire of making more efficient nanoscale electronic devices. In 1974, a theoretical model was suggested for a rectifier made of a single molecule.^1^ This inspiring idea encouraged a significant number of researchers to explore this area of science. Molecular switching is one of the essential search areas as it provides a solution to extremely expand data storage.^2−12^ The current switching devices employ silicon-based technologies, one disadvantage of which is the massive number of silicon atoms involved to save a single signal (i.e., 0 or 1). In comparison, one single magnetic atom was evidenced to perform as a reliable memory, capable of being switched between two magnetic states.^13^

At present, researchers are targeting more complex organic molecules with the goal of obtaining access to multiswitching states than just two (0 and 1), so that the storage capacity expands even further. Multiple molecular switches such as 4 or 14 distinct states^14,15^ satisfy the purpose above. On the other hand, complex molecules typically require advanced and complicated chemistry. One suggestion could be the fullerene derivatives. The possibility of making large hollow carbon cages was suggested in 1960s,^16^ and the existence of the buckminsterfullerene was first predicted in 1970.^17^ After 15 years, the first buckminsterfullerene was discovered by Kroto et al.^18^ One week after the discovery of C_60_, the same group observed the first endohedral metallofullerene, La@C_60_, in the mass spectrum of a sample prepared by laser vaporization of a La@C_l2_-impregnated graphite rod.^19^ A considerable potential advantage of manufacturing electric and thermoelectric devices using endohedral metallofullerenes (EMFs) is their ability to accommodate metallic atoms/moieties inside their cavities.^20−26^

**Figure 1:**
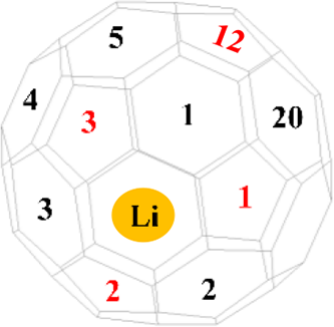
Schematic illustration of the Li@C_60_ complex. Li cation and C_60_ fullerene cage, which possesses 20 hexagonal and 12 pentagonal rings, black and red numbers (for clarity, few numbers are shown).

In the present research, we explore the electronic properties of the Li@C_60_ complex. The major investigation here is dedicated to the electronic structure properties including frontier orbitals, optimization, degeneracy states, charge transfer analyses, and energy difference. These parameters have an essential influence on the electric and thermoelectric transport in the Li@C_60_ complex.^20,27^ To explore the electronic structure of the C_60_ fullerene cage, it should be noted that this spherical cage is built up by hexagon and pentagon rings.^28−31^ It consists of 20 hexagonal and 12 pentagonal rings, as shown in Figure 1. It is widely known that fullerene structures are able to accommodate atom/s inside their empty cavities. Herein, we insert a Li^+^ cation in the C_60_ cage, and the question is where the single alkali metal ion resides in the cavity? Is it static or rattles in the cage?

## 2 Computational Methods

All the theoretical simulations were carried out by employing the DFT code SIESTA.^32^ The optimum geometries of isolated EMF complexes were obtained by relaxing the fullerene cage and its complex until all forces on the atoms were less than 0.01 eV/Å (for more details, see Optimized DFT Structures of Isolated Structures, Supporting Information, and Figure 1). We used a double-zeta plus polarization orbital basis set, norm-conserving pseudopotentials, the local density approximation (LDA) exchange correlation functional, and, to define the real space grid, an energy cutoff of 250 Rydberg. We also computed results using GGA and found that the resulting functions were comparable^33,34^ with those obtained using LDA (note: the generalized gradient approximation (GGA-PBE), of the exchange and correlation functional, was used in this study).

## 3 Results and Discussion

The electronic properties of the C_60_ fullerene cage and Li@C_60_ complex were modeled using a combination of density functional theory (DFT) and quantum transport theory. To have a better understanding of the electronic properties, the frontier orbital of the cage and complex (using Denchar program): highest occupied molecular orbitals (HOMO) and lowest unoccupied orbitals (LUMO), and their extensions (i.e., HOMO–1, HOMO–2,...,HOMO–5), along with their energies, are calculated, as shown in Figures S2 and S3. These isosurface plots clearly demonstrate that the HOMO levels are fivefold degenerate states, while the LUMO orbital levels are triply degenerate states. These results agree with the literature.^35^ Encapsulating the Li cation inside the cage, specifically in the center of the cavity, does not change the degenerate states of the HOMO and LUMO levels, as shown in Supporting Information Figure S3. This result can be explained as follows: insertion of the cation in the center of the C_60_ cage preserves the symmetry of the Li@C_60_ complex, which leads to keeping the degeneracy state unchanged when inserting Li into the spherical cage.

**Figure 2:**
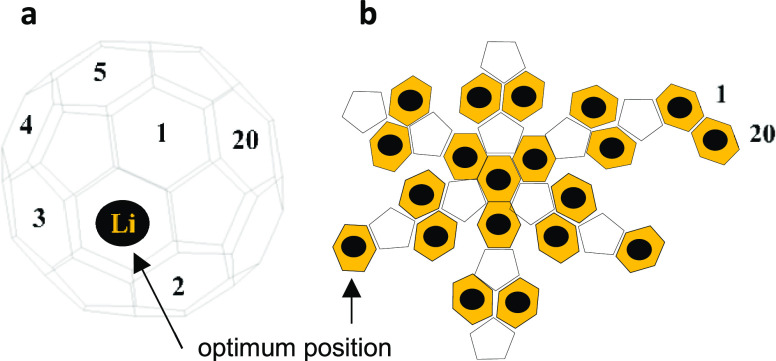
(a) Schematic illustration representing the optimum position of the Li cation inside the C_60_ cage. (b) Deconstructed C_60_ fullerene cage, which shows the 20 optimum positions of Li (hexagonal rings accommodate Li).

To find where the alkali metal sets inside the C_60_ cage, we run 32 different simulations slightly off-center toward the 32 rings (i.e., 20 hexagon and 12 pentagon rings). When the 32 complexes fully optimized lithium, the cation displaces toward one of the 20 hexagonal rings, as shown in Figure 2b. Table S1 shows the off-center displacement for the 20 optimized structures. The off-center varies from 1.2 to 1.4 Å for the 20 relaxed complexes. These figures agree well with the experimental photoelectron and X-ray emission spectral studies,^22,28^ which reported that the Li cation displaces by 1.2 Å from the cavity center (for more details, see the Gas-Phase Relaxations section of the Supporting Information). After finding that the cation does not settle in the cavity center (i.e., 1.2 to 1.4 Å off-center), we repeat the wavefunction calculations to examine the degeneracy status. Figure S5 clearly illustrates that the fivefold degeneracy of HOMOs minimizes to threefold while the threefold LUMO degeneracy is entirely removed. The degeneracy changed because the Li^+^ cation moved from the center of the cage by about 1.2–1.4 Å in 20 off-center displacements, as shown in Table S1. This occurs as the Li@C_60_ complex lost the symmetry when the cation shifted from the cage center.

The next step in exploring the electronic properties of the Li@C_60_ complex is to investigate the charge transfer through this endo complex. The net atomic charge calculation is a regular practice in chemistry measurements and calculations. As a first step, we shall consider charge transfer analyses in gas phase, mainly between the cation and the cage. We assess the net charge transfer from Li toward the C_60_ cage using three different theoretical analyses , namely Mulliken,^36^ Hirshfeld,^37^ and Voronoi.^38^

Table S2 demonstrates that the cation donates electrons to the cage. However, the total number of electrons gained by the 60 carbon atoms depends on the geometrical ring shape, as the cage is made of hexagons and pentagons, and the off-center displacement. We find that the major donation is obtained by six carbons (hexagon ring, C_6_), that face the cation, while a fraction of electron is shared among the rest of the cage atoms (i.e., C_54_). For instance, C_6_ gains 0.186 electrons, whereas C_54_ obtains 0.08 electrons, as in the Mulliken population method. A similar behavior is seen when the Li@C_60_ complex positions on a gold(111) surface; however, the charge migrates from both Li and Au toward the cage (for more details, see the Charge Transfer Analyses of Li@C60 Complex in Gas Phase section of the Supporting Information).

A recent experimental study^15^ reported a 14-state switching behavior of Li@C_60_ complex on an Au(111) substrate using low-temperature ultrahigh vacuum scanning tunneling microscopy and spectroscopy. The current work suggests that the Li cation energetically prefers to be in 20 specific locations within the cage (see Table S1). Building on that, we shall investigate the electrical conductance of the most 20 energetically favorable orientations. To simulate the likely contact configuration during a break-junction experiment, we employed gold metal electrodes constructed from six layers of Au(111), each containing 30 gold atoms, and further terminated with a pyramid of gold atoms. After relaxing each molecular junction (along with PF_6_^–^ as the counterion to keep the whole system natural)^39−41^ with different off-center displacements, varying from 1.2 to 1.4 Å, we calculated the electrical conductance for the 20 most energetically favorable orientations shown in Table S1, using the GOLLUM quantum transport code^42^ (for more details, see the DFT-Based Transport Simulations section in the Supporting Information).

**Figure 3:**
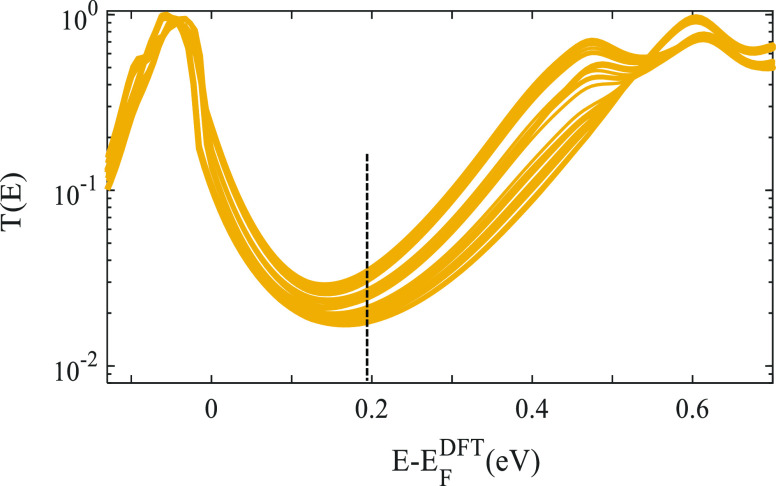
Zero-bias transmission coefficient *T*(*E*) of [Li^+^@C_60_]PF_6_^–^ complex as a function of energy. Twenty orange curves represent the 20 most energetically favorable orientations shown in Table S1. The HOMO resonance is predicted to be pinned near the DFT-predicted Fermi energy; however, the Fermi energy (*E*_F_) is taken in the vicinity of the mid-gap (*E* – *E*_F_^DFT^ ∼ mid-gap).

Recent comparisons between the STM experiment and DFT theory revealed that electron transport through single organic molecules takes place near the middle of the energy gap between the HOMO and LUMO.^12,43−45^ In the current research, indeed, the closest harmonization between theory and experiment is obtained for a Fermi energy (*E*_F_) near the middle of the energy gap (*E****–****E*_F_^DFT^ ∼ mid-gap), as indicated by the vertical dashed line in Figure 3. This figure displays three distinct bands of transmission curves *T*(*E*); we refer to them as high, medium, and low, and each band involves several lines. We attribute the high, medium, and low transmission bands to the three off-center displacements of the Li cation toward the cage, which include 1.4, 1.3, and 1.2 Å (see the Gas-Phase Relaxations section in the Supporting Information). This claim is robustly supported by the conductance value, and the number of transmission curves in each band is as follows: high bands include 6 curves, medium 4, and low 10. These numbers (6, 4, and 10) are equal to the number of off-center displacements. In other words, the high band involves 6× 1.4 Å displacement (i.e., 6 curves with approximately similar conductance values), medium 4× 1.3 Å displacement (i.e., 4 curves), and low 10× 1.2 Å displacement (i.e., 10 curves), as shown in Table S1.

To test the validity of our simulations on the electronic properties of the [Li^+^@C_60_]PF_6_^–^ complex, we shall check it against STM measurements. To perform a decent comparison, we divide the highest conductance states (i.e., 13 and 19 states of STM and DFT, respectively) by the lowest states to obtain switching ratios for both experiment and theory and then compare the corresponding ratios against each other.

**Figure 4:**
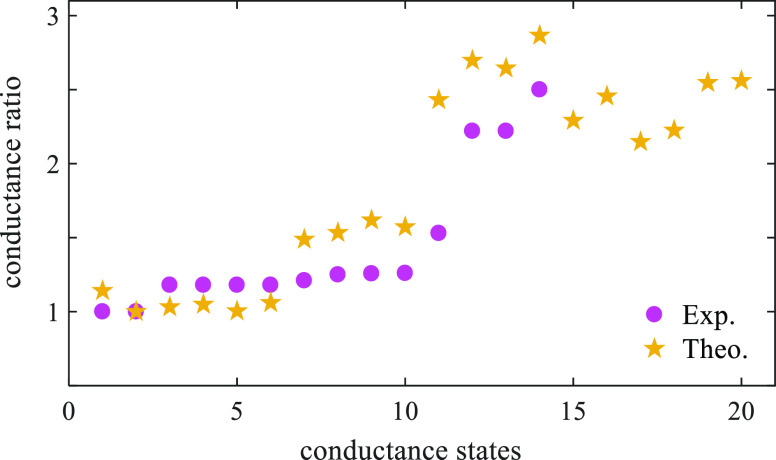
Electrical conductance ratio of [Li^+^@C_60_]PF_6_^–^ complex as a function of switching states, including a comparison between theory and experiment (adapted with permission from ref. 15. Copyright 2019 Nature Communications, 2022). STM experiment comprises 14 switched states (purple circles), while DFT theory involves 20 states (orange stars), which correspond to the 20 most energetically favorable orientations shown in Table S1. Theoretical values are obtained at *E* – *E*_F_^DFT^ ∼ mid-gap.

Figure 4 shows the switching state ratios of DFT simulations and STM measurements. The theoretical predictions of the 20 switching ratios are in broad agreement with the STM measurements. This figure also demonstrates that the on/off switching ratio could reach up to 2.5 experimentally for the [Li^+^@C_60_]PF_6_^–^ complex (state 14), while DFT simulations predict 2.9. This suggests that the DFT simulations are significantly accurate even though STM measurements missed 6 states, which highly expects to bridge the gap even closer.

## 4 Conclusions

In conclusion, through rational simulations, we have demonstrated that the electrical properties of the [Li^+^@C_60_]PF_6_^–^ complex and configurations can be modulated by energetically varying the location of the Li cation that inserts inside the fullerene C_60_ cage. Fully optimized geometries suggest that the cation energetically prefers to be in 20 specific locations within the cage, specifically in the hexagonal rather than pentagonal rings. The geometries also propose that Li displaces away from the center of the spherical cavity, and the off-center displacement varies from 1.2 to 1.4 Å. The off-center displacement violates the degeneracy of the [Li^+^@C_60_]PF_6_^–^ complex; it lowers HOMO’s degeneracy from fivefold to threefold, whereas the threefold LUMO’s degeneracy is completely eliminated.

Charge transfer analyses methods including Mulliken, Hirshfeld, and Voronoi demonstrate the charge migration from a Li cation to a fullerene cage. These analyses specifically indicate that the majority of the transferred charge is gained by the hexagonal ring that faces Li, and a small fraction is distributed on the rest of the cage (C_54_). These findings provide insights into the design and engineering of electrical and thermoelectrical properties. To benchmark our results, we found an excellent correlation of the on/off ratios for multistates between our simulations and STM measurements. This research predicts 20 switching states with an on/off ratio of 2.9, and STM measures 14 states with a ratio of 2.5. This study sheds light on new strategies for designing electronic devices based on tuning the electric structure of cation–cage complexes by using different cations, cages, and orientations with potential practical applications.

## Supporting Information Available

Computational details, optimized structures, frontier orbitals, degeneracy violation, charge transfer analyses, binding energy simulations, and DFT transport calculations (PDF)

## References

[ref1] Aviram A.; Ratner M. A. Molecular rectifiers. J. Chem. Phys. Lett. 1974, 29, 277 10.1016/0009-2614(74)85031-1.

[ref2] González M. T.; Ismael A. K.; Garcia-Iglesias M.; Leary E.; Rubio-Bollinger G.; Grace I.; Gonzalez-Rodriguez D.; Torres T.; Lambert C. J.; Agrait N. Interference Controls Conductance in Phthalocyanine Molecular Junctions. J. Phys. Chem. C 2021, 125, 15035–15043. 10.1021/acs.jpcc.1c03290.

[ref3] Simpson G. J.; Hogan S. W.; Caffio M.; Adams C. J.; Früchtl H.; van Mourik T.; Schaub R. New class of metal bound molecular switches involving H-tautomerism. Nano Lett. 2014, 14, 634–639. 10.1021/nl4038517. 24471795

[ref4] Ye J.; Al-Jobory A.; Zhang Q.-C.; Cao W.; Alshehab A.; Qu K.; Alotaibi T.; Chen H.; Liu J.; Ismael A. K. Highly insulating alkane rings with destructive σ-interference. Sci. China Chem. 2022, 65, 1822–1828. 10.1007/s11426-022-1341-y.

[ref5] Liljeroth P.; Repp J.; Meyer G. Current-induced hydrogen tautomerization and conductance switching of naphthalocyanine molecules. Science 2007, 317, 1203–1206. 10.1126/science.1144366. 17761878

[ref6] Wilkinson L. A.; Bennett T. L.; Grace I. M.; Hamill J.; Wang X.; Au-Yong S.; Ismael A.; Jarvis S. P.; Hou S.; Albrecht T.; Cohen L. F.; Lambert C.; Robinson B. J.; Long N. J. Assembly, structure and thermoelectric properties of 1, 1′-dialkynylferrocene ‘hinges’. Chem. Sci. 2022, 13, 8380–8387. 10.1039/D2SC00861K. 35919728 PMC9297386

[ref7] Huang T.; Zhao J.; Feng M.; Popov A. A.; Yang S.; Dunsch L.; Petek H. A molecular switch based on current-driven rotation of an encapsulated cluster within a fullerene cage. Nano Lett. 2011, 11, 5327–5332. 10.1021/nl2028409. 22081996

[ref8] Bennett T. L.; Alshammari M.; Au-Yong S.; Almutlg A.; Wang X.; Wilkinson L. A.; Albrecht T.; Jarvis S. P.; Cohen L. F.; Ismael A.; Lambert C. J.; Robinson B. J.; Long N. J. Multi-component self-assembled molecular-electronic films: towards new high-performance thermoelectric systems. Chem. Sci. 2022, 13, 5176–5185. 10.1039/D2SC00078D. 35655580 PMC9093172

[ref9] Wang X.; Ismael A.; Ning S.; Althobaiti H.; Al-Jobory A.; Girovsky J.; Astier H. P.; O’Driscoll L. J.; Bryce M. R.; Lambert C. J. Electrostatic Fermi level tuning in large-scale self-assembled monolayers of oligo (phenylene–ethynylene) derivatives. Nanoscale Horiz. 2022, 7, 1201–1209. 10.1039/D2NH00241H. 35913108

[ref10] Ismael A. K.; Lambert C. J. Single-molecule conductance oscillations in alkane rings. J. Mater. Chem. C 2019, 7, 6578–6581. 10.1039/C8TC05565C.

[ref11] Eigler D. M.; Lutz C.; Rudge W. An atomic switch realized with the scanning tunnelling microscope. Nature 1991, 352, 600–603. 10.1038/352600a0.

[ref12] Ismael A.; Al-Jobory A.; Wang X. T.; Alshehab A.; Almutlg A.; Alshammari M.; Grace I.; Benett T. L. R.; Wilkinson L. A.; Robinson B. J.; Long N. J.; Lambert C. Molecular-scale thermoelectricity: as simple as ’ABC’. Nanoscale Adv. 2020, 2, 5329–5334. 10.1039/D0NA00772B. 36132050 PMC9417915

[ref13] Donati F.; Rusponi S.; Stepanow S.; Wäckerlin C.; Singha A.; Persichetti L.; Baltic R.; Diller K.; Patthey F.; Fernandes E.; Dreiser J.; Šljivančanin Ž.; Kummer K.; Nistor C.; Gambardella P.; Brune H. Magnetic remanence in single atoms. Science 2016, 352, 318–321. 10.1126/science.aad9898. 27081065

[ref14] Auwärter W.; Seufert K.; Bischoff F.; Ecija D.; Vijayaraghavan S.; Joshi S.; Klappenberger F.; Samudrala N.; Barth J. V. A surface-anchored molecular four-level conductance switch based on single proton transfer. Nat. Nanotechnol. 2012, 7, 41–46. 10.1038/nnano.2011.211. 22157727

[ref15] Chandler H. J.; Stefanou M.; Campbell E. E.; Schaub R. Li@ C60 as a multi-state molecular switch. Nat. Commun. 2019, 10, 2283 10.1038/s41467-019-10300-2. 31123258 PMC6533348

[ref16] Jones D. Hollow molecules. New Sci. 1966, 32, 245 10.1049/ree.1966.0082.

[ref17] Osawa E. Superaromaticity. Kagaku 1970, 25, 854–863. 10.2307/40201017.

[ref18] Kroto H. W.; Heath J. R.; O’Brien S. C.; Curl R. F.; Smalley R. E. C60: Buckminsterfullerene. Nature 1985, 318, 162–163. 10.1038/318162a0.

[ref19] Kratschmer W.; Lamb L. K. Fostiropoulos and DR Huffman. Nature 1990, 347, 354 10.1038/347354a0.

[ref20] Ismael A. K.; Rincón-García L.; Evangeli C.; Dallas P.; Alotaibi T.; Al-Jobory A. A.; Rubio-Bollinger G.; Porfyrakis K.; Agraït N.; Lambert C. J. Exploring seebeck-coefficient fluctuations in endohedral-fullerene, single-molecule junctions. Nanoscale Horiz. 2022, 7, 616–625. 10.1039/D1NH00527H. 35439804

[ref21] Foroutan-Nejad C.; Andrushchenko V.; Straka M. Dipolar molecules inside C 70: an electric field-driven room-temperature single-molecule switch. Phys. Chem. Chem. Phys. 2016, 18, 32673–32677. 10.1039/C6CP06986J. 27892557

[ref22] Bernshtein V.; Oref I. Surface migrations of endohedral Li+ on the inner wall of C 60. Phys. Rev. A 2000, 62, 033201 10.1103/PhysRevA.62.033201.

[ref23] Raggi G.; Stace A.; Bichoutskaia E. Polarisation charge switching through the motion of metal atoms trapped in fullerene cages. Phys. Chem. Chem. Phys. 2014, 16, 23869–23873. 10.1039/C4CP02672A. 25272966

[ref24] Campbell E. E.; Fanti M.; Hertel I. V.; Mitzner R.; Zerbetto F. The hyperpolarisability of an endohedral fullerene: Li@ C60. Chem. Phys. Lett. 1998, 288, 131–137. 10.1016/S0009-2614(98)00255-3.

[ref25] Al-Khaykanee M. K.; Ismael A. K.; Grace I.; Lambert C. J. Oscillating Seebeck coefficients in π-stacked molecular junctions. RSC Adv. 2018, 8, 24711–24715. 10.1039/C8RA04698K. 35542147 PMC9082453

[ref26] Alshehab A.; Ismael A. K. Impact of the terminal end-group on the electrical conductance in alkane linear chains. RSC Adv. 2023, 13, 5869–5873. 10.1039/D3RA00019B. 36816091 PMC9936266

[ref27] Rincón-García L.; Ismael A. K.; Evangeli C.; Grace I.; Rubio-Bollinger G.; Porfyrakis K.; Agraït N.; Lambert C. J. Molecular design and control of fullerene-based bi-thermoelectric materials. Nat. Mater. 2016, 15, 289–293. 10.1038/nmat4487. 26641017

[ref28] Varganov S.; Avramov P.; Ovchinnikov S. Ab initio calculations of endo-and exohedral C 60 fullerene complexes with Li+ ion and the endohedral C 60 fullerene complex with Li 2 dimer. Phys. Solid State 2000, 42, 388–392. 10.1134/1.1131218.

[ref29] Li Y.; Tománek D. How free are encapsulated atoms in C60?. Chem. Phys. Lett. 1994, 221, 453–458. 10.1016/0009-2614(94)00297-5.

[ref30] Jorn R.; Zhao J.; Petek H.; Seideman T. Current-driven dynamics in molecular junctions: endohedral fullerenes. ACS Nano 2011, 5, 7858–7865. 10.1021/nn202589p. 21882805

[ref31] Ismael A. K.; Lambert C. J. Molecular-scale thermoelectricity: a worst-case scenario. Nanoscale Horiz. 2020, 5, 1073–1080. 10.1039/D0NH00164C. 32432630

[ref32] Soler J. M.; Artacho E.; Gale J. D.; García A.; Junquera J.; Ordejón P.; Sánchez-Portal D. J. J. The SIESTA method for ab initio order-N materials simulation. J. Phys.: Condens. Matter 2002, 14, 2745 10.1088/0953-8984/14/11/302.

[ref33] Davidson R. J.; Milan D. C.; Al-Owaedi O. A.; Ismael A. K.; Nichols R. J.; Higgins S. J.; Lambert C. J.; Yufit D. S.; Beeby A. Conductance of ‘bare-bones’ tripodal molecular wires. RSC Adv. 2018, 8, 23585–23590. 10.1039/C8RA01257A. 35540267 PMC9081744

[ref34] Markin A.; Ismael A. K.; Davidson R. J.; Milan D. C.; Nichols R. J.; Higgins S. J.; Lambert C. J.; Hsu Y.-T.; Yufit D. S.; Beeby A. Conductance Behavior of Tetraphenyl-Aza-BODIPYs. J. Phys. Chem. C 2020, 124, 6479–6485. 10.1021/acs.jpcc.9b10232.

[ref35] Hands I. D.; Dunn J. L.; Bates C. A. Calculation of images of oriented C 60 molecules using molecular orbital theory. Phys. Rev. B 2010, 81, 205440 10.1103/PhysRevB.81.205440.

[ref36] Mulliken R. S. Electronic population analysis on LCAO–MO molecular wave functions. I. J. Chem. Phys. 1955, 23, 1833–1840. 10.1063/1.1740588.

[ref37] Hirshfeld F. L. Bonded-atom fragments for describing molecular charge densities. Theor. Chim. Acta 1977, 44, 129–138. 10.1007/BF00549096.

[ref38] Fonseca Guerra C.; Handgraaf J. W.; Baerends E. J.; Bickelhaupt F. M. Voronoi deformation density (VDD) charges: Assessment of the Mulliken, Bader, Hirshfeld, Weinhold, and VDD methods for charge analysis. J. Comput. Chem. 2004, 25, 189–210. 10.1002/jcc.10351. 14648618

[ref39] Ismael A. K.; Al-Jobory A.; Grace I.; Lambert C. J. Discriminating single-molecule sensing by crown-ether-based molecular junctions. J. Chem. Phys. 2017, 146, 064704 10.1063/1.4975771. 28201900

[ref40] Ismael A. K.; Grace I.; Lambert C. J. Increasing the thermopower of crown-ether-bridged anthraquinones. Nanoscale 2015, 7, 17338–17342. 10.1039/C5NR04907E. 26426840

[ref41] Alshammari M.; Al-Jobory A. A.; Alotaibi T.; Lambert C. J.; Ismael A. Orientational control of molecular scale thermoelectricity. Nanoscale Adv. 2022, 4, 4635–4638. 10.1039/D2NA00515H. 36341305 PMC9595198

[ref42] Ferrer J.; Lambert C. J.; García-Suárez V. M.; Manrique D. Z.; Visontai D.; Oroszlany L.; Rodríguez-Ferradás R.; Grace I.; Bailey S.; Gillemot K.; Sadeghi H.; Algharagholy L. A. GOLLUM: a next-generation simulation tool for electron, thermal and spin transport. New J. Phys. 2014, 16, 093029 10.1088/1367-2630/16/9/093029.

[ref43] Ismael A.; Wang X.; Bennett T. L.; Wilkinson L. A.; Robinson B. J.; Long N. J.; Cohen L. F.; Lambert C. J. Tuning the thermoelectrical properties of anthracene-based self-assembled monolayers. Chem. Sci. 2020, 11, 6836–6841. 10.1039/D0SC02193H. 33033599 PMC7504895

[ref44] Wang X.; Ismael A.; Almutlg A.; Alshammari M.; Al-Jobory A.; Alshehab A.; Bennett T. L.; Wilkinson L. A.; Cohen L. F.; Long N. J. Optimised power harvesting by controlling the pressure applied to molecular junctions. Chem. Sci. 2021, 12, 5230–5235. 10.1039/D1SC00672J. 34163759 PMC8179551

[ref45] Herrer L.; Ismael A.; Martín S.; Milan D. C.; Serrano J. L.; Nichols R. J.; Lambert C.; Cea P. Single molecule vs. large area design of molecular electronic devices incorporating an efficient 2-aminepyridine double anchoring group. Nanoscale 2019, 11, 15871–15880. 10.1039/C9NR05662A. 31414113

