# Supplementary material for: 20-State Molecular Switch in a Li@C60 Complex
Source: ACS Omega. 2023 May 25;8(22):19767–71. doi: 10.1021/acsomega.3c01455 (PMC10249121; doi:10.1021/acsomega.3c01455)
Supplement: Supplementary file 1 — ao3c01455_si_001.pdf [file ao3c01455_si_001.pdf]

Supporting Information

A 20-state molecular switch in Li@C_{60} complex

Ali K. Ismael[a,b*]

[a]Department of Physics, Lancaster University, Lancaster LA1 4YB, UK.
[b]Department of Physics, College of Education for Pure Science, Tikrit University, Tikrit, Iraq.

Table of contents

Computational details 2
1. Optimised DFT Structures of Isolated Moieties 2
2. Frontier orbitals of the studied EMFs complexes 2
3. Gas-phase relaxations…………..6
4. Degeneracy violation…………...7
5. Charge transfer analyses…………....................9
6. Binding energies of EMFs on a gold surface 11
7. DFT-based transport simulations…………12

References....................13

Computational details.

1. Optimised DFT structures of isolated structures

Using the density functional code SIESTA,[1, 2] the optimum geometries of the isolated moieties including C_{60} fullerene cage and Li@C_{60} complex were obtained by relaxing the molecules until all forces on the atoms were less than 0.01 eV / Å as shown in Fig. SI.1. A double-zeta plus polarization orbital basis set, norm-conserving pseudopotentials, with an energy cut-off of 250 Rydbergs, defined on the real space grid was used and the local density approximation (GGA) was chosen to be the exchange correlation functional.

a b

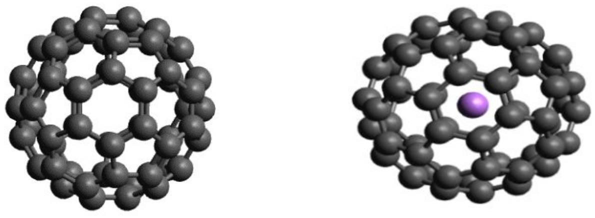

Figure S1. Geometries of the isolated studied molecules. (**a**) C_{60} spherical fullerene cage. (**b**) Li@C_{60} complex.

2. Frontier orbitals of the studied structures

To obtain a better understanding of the electronic properties of these structures (see Figure S1), we will investigate the wave function plots of the empty cage C_{60} and Li@C_{60} complex. The highest occupied molecular orbitals (HOMO), lowest unoccupied orbitals (LUMO), and their extensions (i.e., HOMO+1, HOMO+2… etc), along with their energies are calculated. The blue and red colours correspond to the regions in space of positive and negative orbital amplitude. Figures S2-S3 show the frontier orbitals of the studied systems, after relaxing the structures until all forces on the atoms were less than 0.01 eV/Å. The local density approximation (LDA) was chosen to be the exchange correlation functional. We also computed results using GGA and found that the results were comparable with those obtained using LDA.[3-5] Figures S2-S3 illustrate the theoretical frontier orbitals of the isolated structures. DFT tends to underestimate the HOMO-LUMO gap,[6, 7] which is why the calculated gaps S1 are smaller than the optically-measured gaps reported in.[8]

2.1. Frontier orbitals of the empty cage

E_F = -3.90 eV

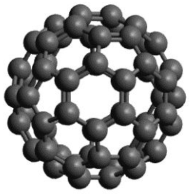

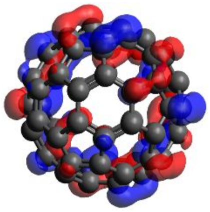

HOMO= -4.61 eV

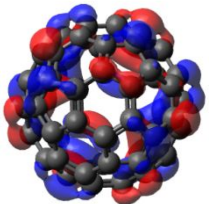

LUMO=- 3.25 eV

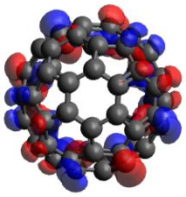

HOMO-1= -4.61 eV

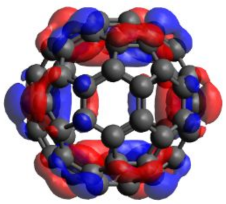

LUMO+1=-3.24 eV

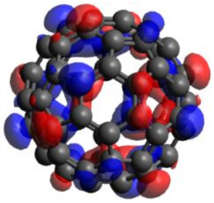

HOMO-2= -4.62 eV

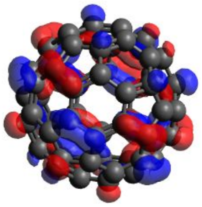

LUMO+2= -3.24 eV

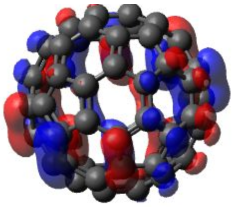

HOMO-3= - 4.63eV

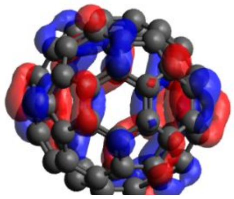

LUMO+3=-2.12 eV

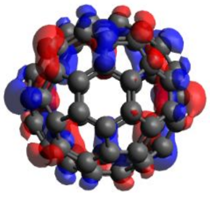

HOMO-4=-4.63 eV

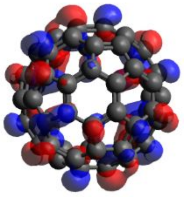

LUMO+4=-2.12 eV

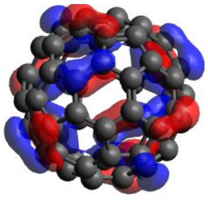

HOMO-5= -5.81 eV

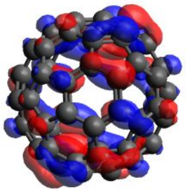

LUMO+5= -2.11 eV

Figure S2. Wave function plots of C_{60} cage. **Top panel**: fully optimised geometry of C_{60} fullerene. **Lower panel**: HOMOs and LUMOs orbitals of C_{60} cage along with their energies. Five-fold degeneracy for HOMO levels and three-fold degeneracy for LUMO levels.

Figure S2, demonstrates that the HOMO orbital levels are fivefold degenerate states while LUMO orbital levels are triply degenerate states. These results fit with the literature.[9] Encapsulating the Li cation inside the cage, specifically in the centre of the cavity is not changing the degenerate states of the HOMO and LUMO orbital levels as shown in Figure S3. This result can be explained as inserting the cation in the centre of C_{60} cage preserves the symmetry of Li@C_{60} complex (i.e., spherical cage + Li).

2.2. Frontier orbitals of Li@C_{60} complex

E_F = -3.30 eV

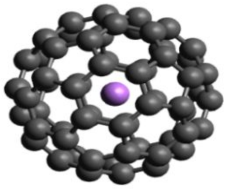

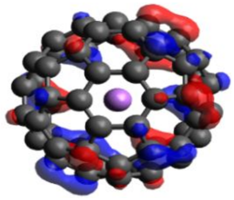

HOMO= -4.83 eV

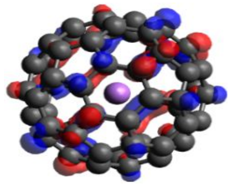

LUMO=- 3.27 eV

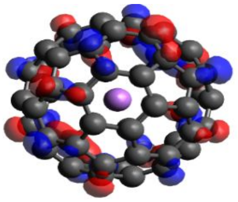

HOMO-1= -4.83 eV

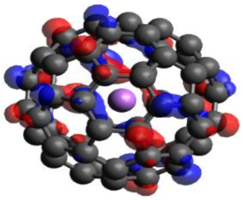

LUMO+1=-3.27 eV

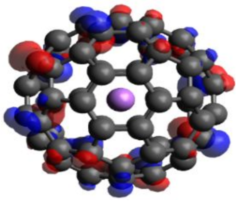

HOMO-2= -4.84 eV

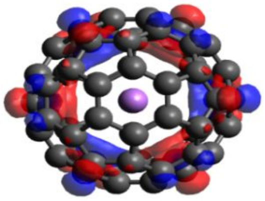

LUMO+2= -3.27 eV

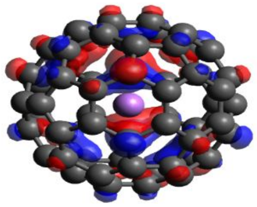

HOMO-3= - 4.85eV

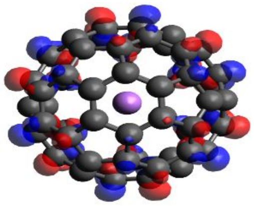

LUMO+3=-2.17 eV

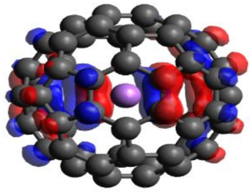

LUMO-4=-4.85 eV

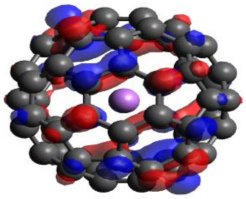

LUMO+4=-2.15 eV

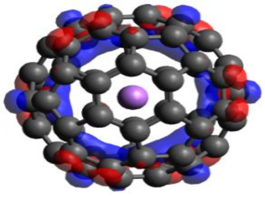

HOMO-5= -5.97 eV

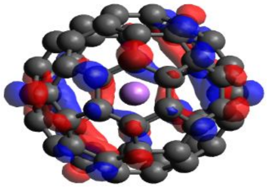

LUMO+5= -2.14 eV

Figure S3. Wave function plots of Li@C_{60} complex. **Top panel**: fully optimised geometry of Li@C_{60} complex. **Lower panel**: HOMOs and LUMOs orbitals of Li@C_{60} complex along with their energies. Five-fold degeneracy for HOMO levels and three-fold degeneracy for LUMO levels.

3. Gas-phase relaxations

To find where the Li cation sets inside the C_{60} cage, we run 32 different simulations slightly off-centre towards the 32 rings (i.e., 20 hexagon and 12 pentagon rings). When the 32 complexes fully optimised the lithium, cation displaces towards one of the 20 hexagonal rings as shown in Fig. S4.

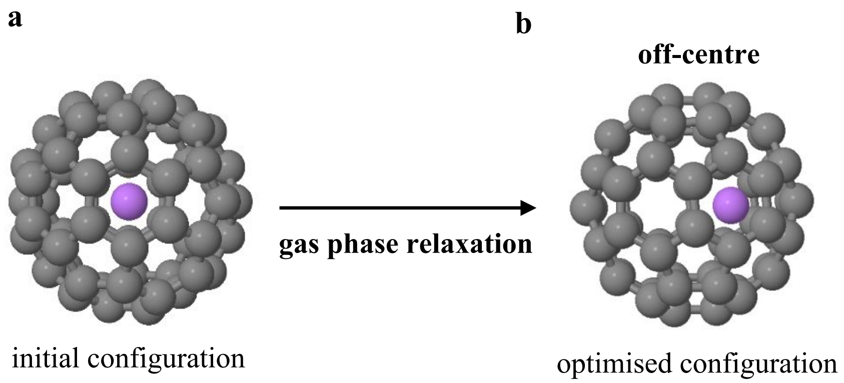

Figure S4. Fully optimised geometry of Li@C_{60} complex. (**a**) Li cation at the centre of spherical fullerene cage. (**b**) Li cation displaces off-centre of the Li@C_{60} complex.

Table S1, shows off-centre displacement for the 20 optimisation strictures. This table illustrates that the off-centre varies from 1.2 to 1.4 Å for the 20 relaxed complexes. These figures agree well with the experimental photoelectron and X-ray emission spectra reported study,[10] that found the Li cation displaces by 1.2 Å.

Table S1. Summarises the off-centre displacement of 20 fully optimised Li@C_{60} complex structures in Å unit.

ring number	off-entre displacement (Å)	ring number	off-entre displacement (Å)
**1**	1.4	**11**	1.2
**2**	1.2	**12**	1.4
**3**	1.3	**13**	1.2
**4**	1.3	**14**	1.2
**5**	1.2	**15**	1.4
**6**	1.2	**16**	1.2
**7**	1.4	**17**	1.3
**8**	1.2	**18**	1.3
**9**	1.2	**19**	1.2
**10**	1.4	**20**	1.4

4. Degeneracy violation

Gas-phase relaxations above, suggest the lithium cation energetically prefers to be in 20 specific locations within the fullerene cage, specifically in hexagonal rather than pentagonal rings. These also propose the Li displaces away from the centre of the spherical cavity and off-centre displacement varies from 1.2 – 1.4 Å. Off-centre displacement violates the five-fold degeneracy of Li@C60 complex as shown in Fig S5. The five-fold degeneracy of HOMO levels reduces to three while three-fold degeneracy of LUMO levels has completely lifted.

Figure S5. Wave function plots of $Li@C_{60}$ complex at off-centre displacement. **Top panel**: fully optimised geometry of $Li@C_{60}$ complex. **Lower panel**: HOMOs and LUMOs orbitals of $Li@C_{60}$ complex along with their energies. Three-fold degeneracy for HOMO levels and zero degeneracy for LUMO levels.

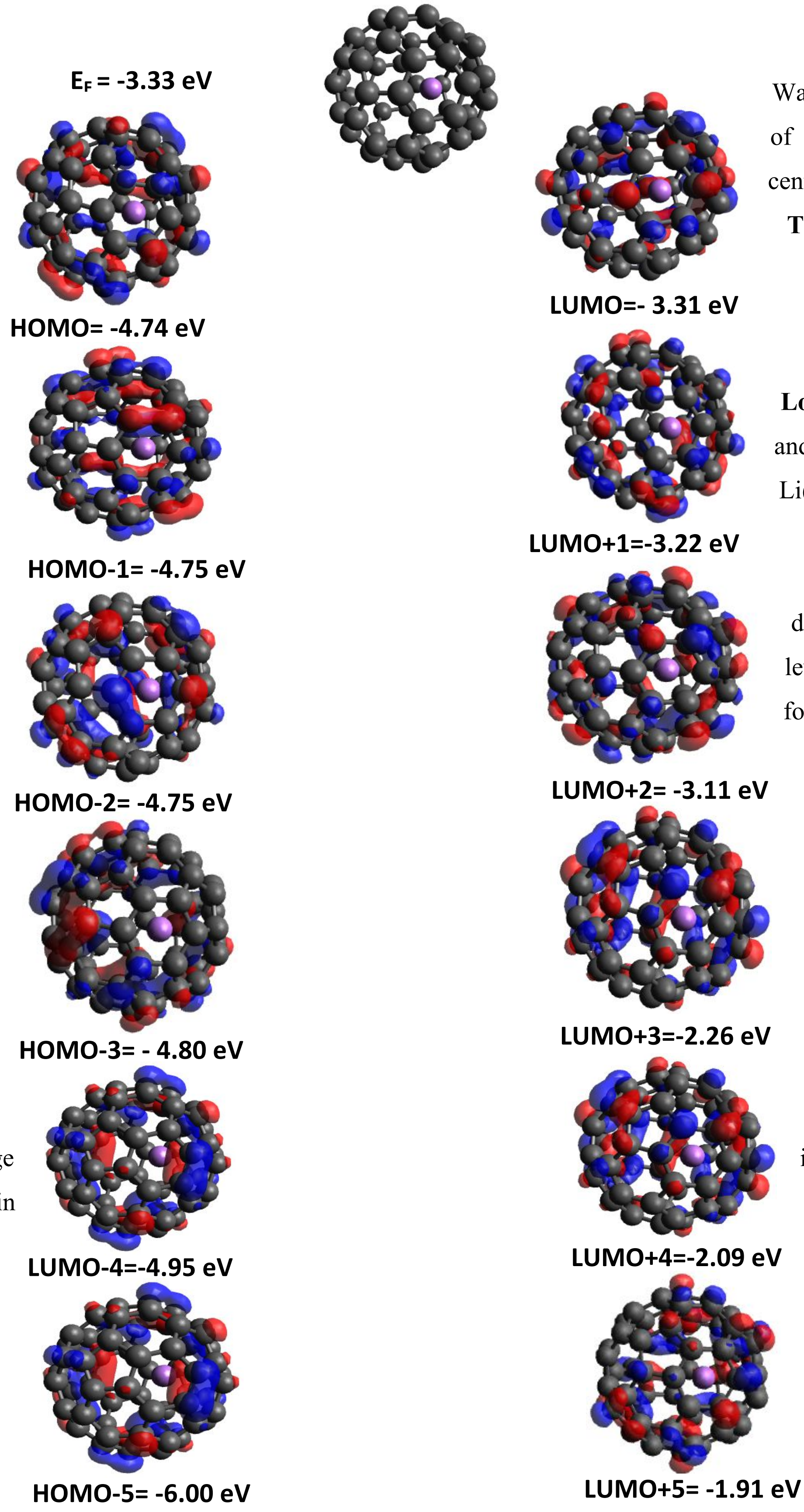

5. Charge transfer analyses

Net atomic charge is a common idea in all chemical sciences. It is difficult to imagine learning chemistry without discussing net atomic

charges.[11] For example, experiments measuring the water molecule's dipole moment imply a negative net atomic charge on its oxygen atom and a positive net atomic charge on each of its two hydrogen atoms.[12] Net atomic charge also plays an important role in solid state physics, where oxygen atoms in solid oxides carry negative net atomic charges to enable oxygen ion transport.[13] There are many methods to calculate the charge transfer in Density Functional Theory. In this chapter, I am going to focus on three methods, that are implemented in SIESTA code, including Mulliken populations, Hershfield and Voronoi charge analyses.

Here, we will investigate the electrical properties of Li@C_{60} complex first in the gas phase, then we repeat the same calculations, but on an Au substrate. Electrons are expected to be transferred from the donor moiety (Li cation) to the acceptor (the cage). The three methods Mulliken, Hirshfeld and Voronoi will be used to determine the charge transfer from the donor to the acceptor.

5.1. Charge transfer analyses of Li@C_{60} complex in gas phase

Tables S2, show, the charge transfer from the Li cation to the C_{60} cage. The second row of Table S2, illustrates that the Li loses (+) in total 0.266 electrons. 0.186 is the net charge that has been gained (-) by the hexagonal ring that faces the Li cation and 0.080 by the rest of the cage (C_{54}), these figures estimated by the Mulliken method. Hirshfeld and Voronoi charge analyse follow a similar trend; the net charges are 0.272 and 0.295 electrons respectively. It is worth mentioning that, the charge transferred from the cation to the cage and the charge effected on the electrical conductance G and Seebeck coefficients S.

Table S2: Gas phase charge transfer analyses using Mulliken, Hirshfeld and Voronoi methods of Li@C_{60} complex. The total number of electrons transferred from Li cation (with a charge of +|e|), to C_{60} cages (with a charge of -|e|), to form a complex.

Element	Mulliken charge		Hirshfeld charge		Voronoi charge	
	Li	C_{60} cage	Li	C_{60} cage	Li	C_{60} cage
**C1**	+0.266	-0.029	+0.272	-0.028	+0.295	-0.031
**C2**		-0.033		-0.031		-0.034
**C3**		-0.032		-0.028		-0.033
**C4**		-0.031		-0.027		-0.032
**C5**		-0.028		-0.026		-0.030
**C6**		-0.033		-0.028		-0.031

**C6_tot**		-0.186		-0.168		-0.192
**C54_tot**		-0.080		-0.104		-0.103
**Li**	+0.266		+0.272		+0.295	

5.2. Charge transfer analyses of Li@C_{60} complex on a gold (111), surface

The analyses here are built on the 3 factors, namely the Li, the cage and a gold substrate. Table S3, shows the amount of charge transfer from the Li cation and Au substrate to C_{60} cage. The second column of Table S3, illustrates that the Li and Au lose (+) in total 0.312 electrons. 0.282 is the net charge that has been gained (-) by the hexagonal ring and the rest by the 54 atoms of the cage, these figures estimated by the Mulliken method. Hirshfeld and Voronoi charge analyse follow a similar trend.

Table S3: Charge transfer analyses using Mulliken, Hirshfeld and Voronoi methods of Li@C_{60} complex. The total number of electrons transferred from Li cation and Au surface (with a charge of +|e|, numbers in bracket for Au), to C_{60} cages (with a charge of -|e|), to form a complex on an Au (111), surface.

Element	Mulliken charge		Hirshfeld charge		Voronoi charge	
	**Li + Au**	**C_{60} cage**	**Li + Au**	**C_{60} cage**	**Li + Au**	**C_{60} cage**
**C1**	+0.251	-0.041	+0.263	-0.018	+0.276	-0.041
**C2**	(+0.061)	-0.044	(+0.002)	-0.017	(+0.006)	-0.032
**C3**		-0.094		-0.032		-0.033
**C4**		-0.050		-0.031		-0.028
**C5**		-0.029		-0.025		-0.026
**C6**		-0.024		-0.033		-0.030
**C6_tot**		-0.282		-0.156		-0.177
**C54_tot**		-0.030		-0.109		-0.105
**(Li+Au)_tot**	+0.312		+0.265		+0.282	

6. Binding energies of Li@C_{60} complex on a gold surface

To calculate the optimum binding distance for Li@C_{60} complex on the gold (111) surface, we use DFT, combined with the counterpoise method, which removes basis set superposition errors (BSSE). As shown by the example of Li@C_{60} in Figure S6, the distance z is defined as the distance between the Au surface and the nearest C atom of the C_{60} cage (see the black double-arrow on the right panel of Figure S6).

The ground state energy of the total system is calculated using SIESTA[1] and is denoted E_{AB}^{AB}. Here the gold leads consist of 3 layers of 25 atoms. The Li@C_{60} complex is defined as monomer A and the gold electrodes as monomer B. The binding energy of each molecule is then calculated in a fixed basis, which is achieved through the use of ghost atoms in SIESTA. Hence the energy of the isolated Li@C_{60} in the presence of the fixed basis is defined as E_A^{AB} and for the isolated gold is E_B^{AB}. The energy difference ($\Delta(z)$) between the isolated entities and their total energy when placed a distance z apart is then calculated using the following equation:

$$\text{Energy difference } = \Delta(z) = E_{AB}^{AB}(z) - E_A^{AB} - E_B^{AB} \qquad \text{(S1)}$$

We repeat the same technique for the 20 energetically-favourable orientations as illustrated in Table S1. As shown by the Figure S5, the equilibrium distance for Li@C_{60}, corresponding to the minimum energy difference, is found to be approximately 2.5 Å, which is comparable with a value of 2.4 Å reported in Ref. [14].

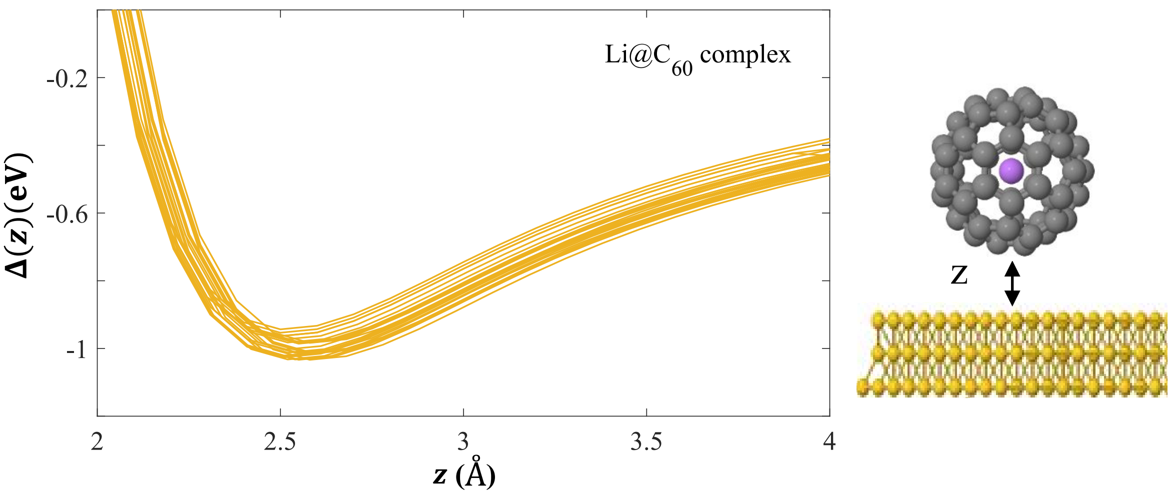

Figure S6. Li@C_{60} complex on a (111) gold surface (Right panel). Energy difference of Li@C_{60} /gold complex as a function of complex-gold distance for the 20 most energetically-favourable orientations (orange curves). The equilibrium distance corresponding to the energy minimum is found to be approximately 2.5 Å (Left panel).

7. DFT-based transport simulations

In the following transport calculations, the ground-state Hamiltonian and optimised geometry of the 20 complexes were obtained using the density-functional theory (DFT) code.[1] The local-density approximation (LDA) exchange-correlation functional was used along with double-zeta-polarized (DZP) basis sets and the norm-conserving pseudo-potentials. The real-space grid was defined by a plane-wave cut-off of 250 Ry. Geometry optimization was carried out to a force tolerance of 0.01 eV/Å. This process was repeated for a unit cell with the molecule between gold electrodes where the optimised distances between complexes and Au electrodes are shown in Fig. S6. From the ground-state Hamiltonian, the transmission coefficient, and hence the room-temperature electrical conductance G, were obtained as described in the sections below. We modelled the properties of a single molecule in the junction.

To calculate the electrical transport through Li@C_{60} complexes along with a corresponding counterion (PF_6^-), we modelled the Au-terminal junction. The gold electrode is modelled as a nanowire attached to each molecule. A mean-field Hamiltonian and an overlap matrix were extracted from the converged DFT calculation and combined with our quantum-transport calculation code, Gollum, to calculate the transmission coefficient T(E) of electrons of energy E passing from the one Au electrode to another through the Li@C_{60} complexes as shown in Fig. S7.

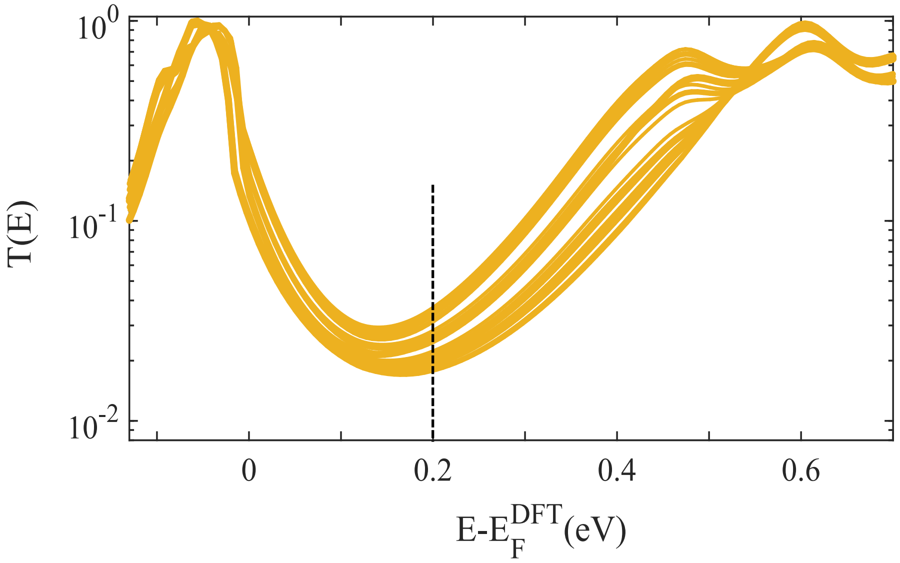

Figure S7. Zero-bias transmission coefficient T(E) of Li@C_{60} complex as a function of energy. 20 orange curves represent the 20 most energetically-favourable orientations that shown in Table S1. The HOMO resonance is predicted to be pinned near the DFT-predicted Fermi energy.

References

1. Soler, J. M.; Artacho, E.; Gale, J. D.; García, A.; Junquera, J.; Ordejón, P.; Sánchez-Portal, D. J. J. o. P. C. M., The SIESTA method for ab initio order-N materials simulation. *Journal of Physics: Condensed Matter* **2002,** *14* (11), 2745.

2. Emilio, A.; Anglada, E.; Diéguez, O.; Gale, J. D.; García, A.; Junquera, J.; Martin, R. M.; Ordejón, P.; Pruneda, J. M.; Sánchez-Portal, D.; Soler, J. M., The SIESTA method; developments and applicability. *J. Phys.: Condens. Matter* **2008,** *20* (6), 064208.

3. Herrer, I. L.; Ismael, A. K.; Milan, D. C.; Vezzoli, A.; Martín, S.; González-Orive, A.; Grace, I.; Lambert, C.; Serrano, J. L.; Nichols, R. J., Unconventional single-molecule conductance behavior for a new heterocyclic anchoring group: pyrazolyl. *The journal of physical chemistry letters* **2018,** *9* (18), 5364-5372.

4. Ismael, A. K.; Wang, K.; Vezzoli, A.; Al-Khaykanee, M. K.; Gallagher, H. E.; Grace, I. M.; Lambert, C. J.; Xu, B.; Nichols, R. J.; Higgins, S. J., Side-Group-Mediated Mechanical Conductance Switching in Molecular Junctions. *Angewandte Chemie International Edition* **2017,** *56* (48), 15378-15382.

5. Markin, A.; Ismael, A. K.; Davidson, R. J.; Milan, D. C.; Nichols, R. J.; Higgins, S. J.; Lambert, C. J.; Hsu, Y.-T.; Yufit, D. S.; Beeby, A., Conductance Behavior of Tetraphenyl-Aza-BODIPYs. *The Journal of Physical Chemistry C* **2020,** *124* (12), 6479-6485.

6. Lof, R.; Van Veenendaal, M.; Koopmans, B.; Jonkman, H.; Sawatzky, G., Band gap, excitons, and Coulomb interaction in solid C 60. *Physical review letters* **1992,** *68* (26), 3924.

7. Hung, Y.-C.; Jiang, J.-C.; Chao, C.-Y.; Su, W.-F.; Lin, S.-T., Theoretical Study on the Correlation between Band Gap, Bandwidth, and Oscillator Strength in Fluorene-Based Donor– Acceptor Conjugated Copolymers. *The Journal of Physical Chemistry B* **2009,** *113* (24), 8268-8277.

8. Ismael, A. K.; Rincón-García, L.; Evangeli, C.; Dallas, P.; Alotaibi, T.; Al-Jobory, A. A.; Rubio-Bollinger, G.; Porfyrakis, K.; Agraït, N.; Lambert, C. J., Exploring seebeck-coefficient fluctuations in endohedral-fullerene, single-molecule junctions. *Nanoscale Horizons* **2022,** *7*, 616-625.
9. Hands, I. D.; Dunn, J. L.; Bates, C. A., Calculation of images of oriented C 60 molecules using molecular orbital theory. *Physical Review B* **2010,** *81* (20), 205440.
10. Varganov, S.; Avramov, P.; Ovchinnikov, S., Ab initio calculations of endo-and exohedral C 60 fullerene complexes with Li+ ion and the endohedral C 60 fullerene complex with Li 2 dimer. *Physics of the Solid State* **2000,** *42*, 388-392.
11. Shusterman, A. J.; Hoistad, L. M., Teaching Chemistry with Electron Density Models. 2. Can Atomic Charges Adequately Explain Electrostatic Potential Maps? *The Chemical Educator* **2001,** *6* (1), 36-40.
12. Clough, S. A.; Beers, Y.; Klein, G. P.; Rothman, L. S., Dipole moment of water from Stark measurements of H2O, HDO, and D2O. *The Journal of Chemical Physics* **1973,** *59* (5), 2254-2259.
13. Skinner, S. J.; Kilner, J. A., Oxygen ion conductors. *Materials Today* **2003,** *6* (3), 30-37.
14. Rincón-García, L.; Ismael, A. K.; Evangeli, C.; Grace, I.; Rubio-Bollinger, G.; Porfyrakis, K.; Agraït, N.; Lambert, C. J., Molecular design and control of fullerene-based bi-thermoelectric materials. *Nature materials* **2016,** *15* (3), 289-293.
